# *catena*-Poly[[(dimethyl sulfoxide-κ*O*)zinc(II)]-μ-(*E*)-2-[(2-oxido-1-naphth­yl)­methyl­eneamino]propanoato-κ^4^ *O* ^2^,*N*,*O* ^1^:*O* ^1′^]

**DOI:** 10.1107/S1600536808030614

**Published:** 2008-09-27

**Authors:** Jun You, Bo Liu, Yan-Qiong Chen, Cui Xiao, Duo-Qi Zhai

**Affiliations:** a School of Chemistry and Environment Engineering, Harbin University of Science and Technology, Harbin 150040, People’s Republic of China

## Abstract

In the title coordination polymer, [Zn(C_14_H_11_NO_3_)(C_2_H_6_OS)]_*n*_, each Zn^II^ ion is five-coordinated in a slightly distorted trigonal–bipyramidal coordination environment, formed by three O atoms from two 2-[(2-oxido-1-naphth­yl)­methyl­eneamino]propanoate ligands, one O atom from a dimethyl sulfoxide mol­ecule and the N atom from the amino­propanoate ligand. The propanoate ligands bridge Zn^II^ ions, forming a zigzag chain parallel to [010].

## Related literature

For the synthesis of (*E*)-2-[(2-hydroxy­naphthalen-1-yl)­methyl­eneamino]propanoic acid, see: Audriceth *et al.* (1954 ▶).
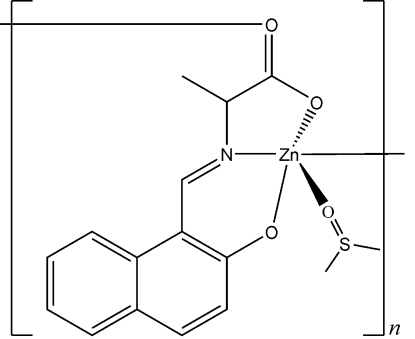


## Experimental

### 

#### Crystal data

[Zn(C_14_H_11_NO_3_)(C_2_H_6_OS)]
*M* *_r_* = 384.74
Monoclinic, 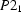

*a* = 9.676 (4) Å
*b* = 7.651 (4) Å
*c* = 11.715 (5) Å
β = 106.256 (15)°
*V* = 832.6 (7) Å^3^
*Z* = 2
Mo *K*α radiation
μ = 1.62 mm^−1^
*T* = 291 (2) K
0.31 × 0.28 × 0.24 mm

#### Data collection

Rigaku R-AXIS RAPID diffractometer
Absorption correction: multi-scan (*ABSCOR*; Higashi, 1995 ▶) *T*_min_ = 0.633, *T*_max_ = 0.697
8191 measured reflections
3729 independent reflections
3533 reflections with *I* > 2σ(*I*)
*R*_int_ = 0.020

#### Refinement

*R*[*F* ^2^ > 2σ(*F* ^2^)] = 0.024
*wR*(*F* ^2^) = 0.056
*S* = 1.06
3729 reflections
211 parameters
1 restraint
H-atom parameters constrained
Δρ_max_ = 0.25 e Å^−3^
Δρ_min_ = −0.19 e Å^−3^
Absolute structure: Flack (1983 ▶), 1675 Friedel pairs
Flack parameter: 0.006 (8)

### 

Data collection: *RAPID-AUTO* (Rigaku, 1998 ▶); cell refinement: *RAPID-AUTO*; data reduction: *CrystalStructure* (Rigaku/MSC, 2002 ▶); program(s) used to solve structure: *SHELXS97* (Sheldrick, 2008 ▶); program(s) used to refine structure: *SHELXL97* (Sheldrick, 2008 ▶); molecular graphics: *SHELXTL* (Sheldrick, 2008 ▶); software used to prepare material for publication: *SHELXL97*.

## Supplementary Material

Crystal structure: contains datablocks I, global. DOI: 10.1107/S1600536808030614/ng2495sup1.cif

Structure factors: contains datablocks I. DOI: 10.1107/S1600536808030614/ng2495Isup2.hkl

Additional supplementary materials:  crystallographic information; 3D view; checkCIF report

## Figures and Tables

**Table:** 

N1—Zn1	2.0119 (18)
O1—Zn1	2.0040 (15)
O2—Zn1	2.1891 (16)
O3—Zn1^i^	1.9560 (15)
O4—Zn1	2.0520 (17)

**Table:** 

O3^ii^—Zn1—O1	98.54 (7)
O3^ii^—Zn1—N1	138.57 (7)
O1—Zn1—N1	88.32 (7)
O3^ii^—Zn1—O4	101.12 (7)
O1—Zn1—O4	96.09 (7)
N1—Zn1—O4	118.82 (7)
O3^ii^—Zn1—O2	93.89 (7)
O1—Zn1—O2	166.10 (6)
N1—Zn1—O2	78.22 (6)
O4—Zn1—O2	87.52 (7)

Symmetry codes: (i) 
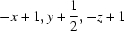
; (ii) 
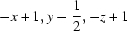
.

**Fig. 1.**
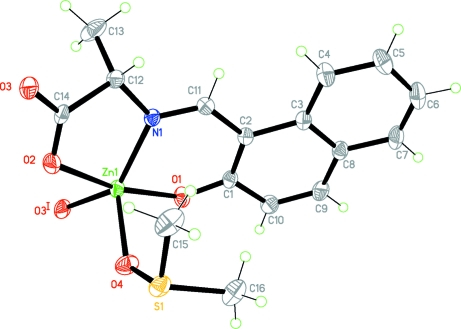
The molecular structure of (I), showing displacement ellipsoids at the 30% probability level for non-H atoms. [Symmetry code: (I) -x + 1, 1/2 + y, -z + 1].

**Fig. 2.**
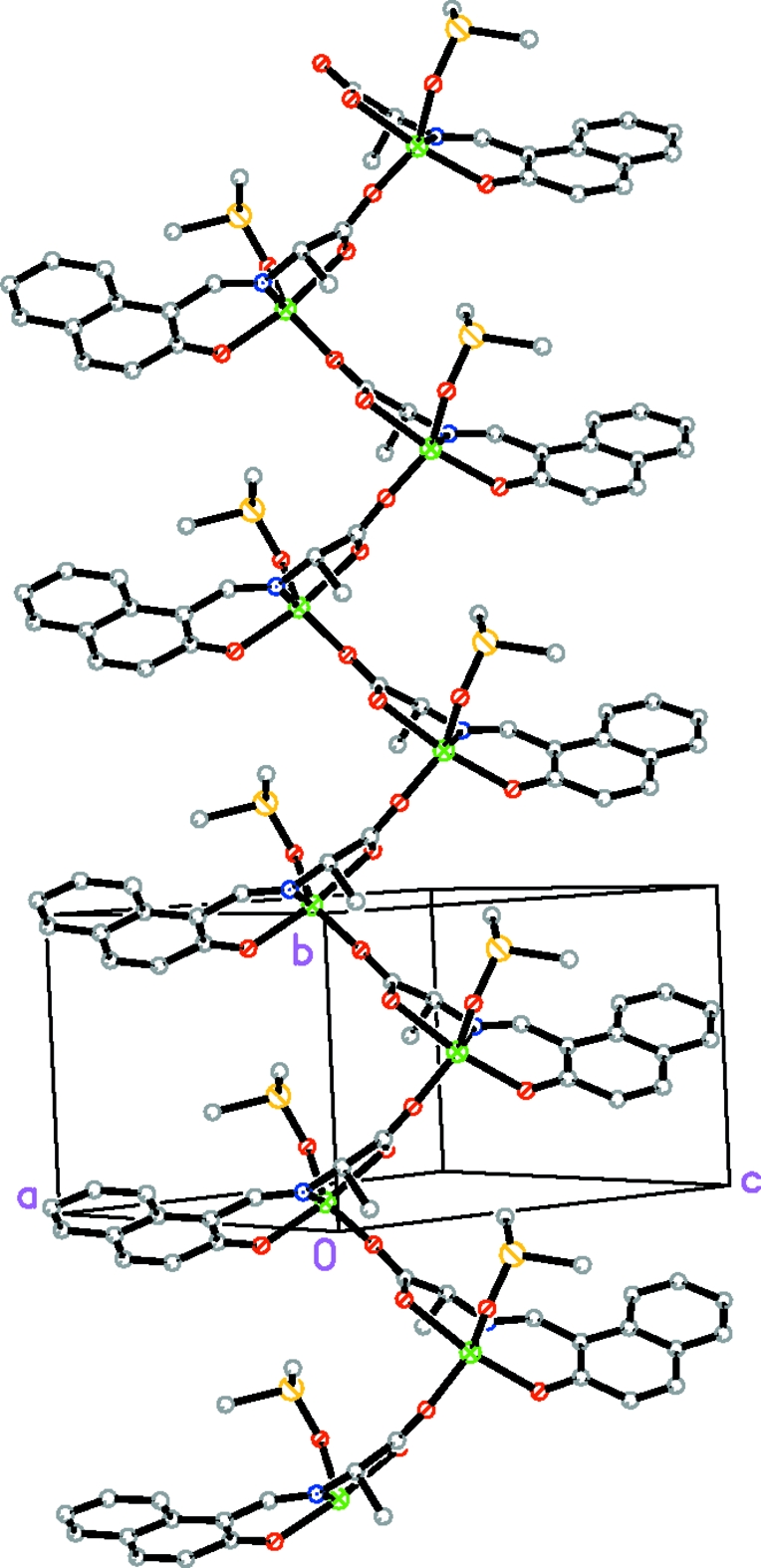
A partial packing view, showing the one-dimensional chain structure. H atoms have been omitted for clarity.

## supplementary crystallographic information

### Comment

The continuous interest in designing and making novel Schiff base ligand and transition-metal complexes have persisted because of their impressive catalytic property. In this paper, we report the new title compound, (I), synthesized by the reaction of (*E*)-2-((2-hydroxynaphthalen-1-yl)methyleneamino)propanoic acid ligands and Zn(OAc)_2_ in an aqueous solution.

As shown in Fig. 1, Zn^II^ ion is five-coordinate in a slightly distorted trigonal-bipyramidal coordination environment, formed by four O atoms and one N atom. Each quadridentate Schiff base ligand bridge two different Cu^II^ ions, resulting in a one-dimensional polymeric structure chain (Fig. 2).

### Experimental

(*E*)-2-((2-Hydroxynaphthalen-1-yl)methyleneamino)propanoic acid was prepared of *L*-alanine acid and 2-hydroxy-1-naphthaldehyde in aqueous solution (Audriceth *et al.*, 1954). (*E*)-2-((2-Hydroxynaphthalen-1-yl)methyleneamino)propanoic acid (0.243 g, 1 mmol) and Zn(OAc)_2_ (0.190 g, 1 mmol) dissolved in hot aqueous solution (20 ml) then refluxed for 1 huor. After cooling to room temperature the solution was filtered, the residue was recrystaled in DMSO and methanol (10/1, V/V) solution, several days latter, a suitable for X-ray diffraction yellow crystal was obtained.

### Refinement

H atoms bound to C atoms were placed in calculated positions and treated as riding on their parent atoms, with C—H = 0.93 Å (Caromatic); C—H = 0.96 Å (methyl); C—H = 0.98 ° A (methine), and with *U*_iso_(H) = 1.2*U*_eq_(C).

### Crystal data

**Table:**

[Zn(C_14_H_11_NO_3_)(C_2_H_6_OS)]	*F*(000) = 396
*M**_r_* = 384.74	*D*_x_ = 1.535 Mg m^−^^3^
Monoclinic, *P*2_1_	Mo *K*α radiation, λ = 0.71073 Å
Hall symbol: P 2yb	Cell parameters from 7870 reflections
*a* = 9.676 (4) Å	θ = 3.2–27.5°
*b* = 7.651 (4) Å	µ = 1.62 mm^−^^1^
*c* = 11.715 (5) Å	*T* = 291 K
β = 106.256 (15)°	Block, yellow
*V* = 832.6 (7) Å^3^	0.31 × 0.28 × 0.24 mm
*Z* = 2	

### Data collection

**Table:**

Rigaku R-AXIS RAPID diffractometer	3729 independent reflections
Radiation source: fine-focus sealed tube	3533 reflections with *I* > 2σ(*I*)
graphite	*R*_int_ = 0.020
ω scans	θ_max_ = 27.5°, θ_min_ = 3.2°
Absorption correction: multi-scan (ABSCOR; Higashi, 1995)	*h* = −11→12
*T*_min_ = 0.633, *T*_max_ = 0.697	*k* = −9→9
8191 measured reflections	*l* = −15→15

### Refinement

**Table:**

Refinement on *F*^2^	Secondary atom site location: difference Fourier map
Least-squares matrix: full	Hydrogen site location: inferred from neighbouring sites
*R*[*F*^2^ > 2σ(*F*^2^)] = 0.024	H-atom parameters constrained
*wR*(*F*^2^) = 0.056	*w* = 1/[σ^2^(*F*_o_^2^) + (0.0259*P*)^2^] where *P* = (*F*_o_^2^ + 2*F*_c_^2^)/3
*S* = 1.06	(Δ/σ)_max_ = 0.001
3729 reflections	Δρ_max_ = 0.25 e Å^−^^3^
211 parameters	Δρ_min_ = −0.19 e Å^−^^3^
1 restraint	Absolute structure: Flack (1983), 1675 Friedel pairs
Primary atom site location: structure-invariant direct methods	Flack parameter: 0.006 (8)

### Special details

**Table:**

Geometry. All e.s.d.'s (except the e.s.d. in the dihedral angle between two l.s. planes) are estimated using the full covariance matrix. The cell e.s.d.'s are taken into account individually in the estimation of e.s.d.'s in distances, angles and torsion angles; correlations between e.s.d.'s in cell parameters are only used when they are defined by crystal symmetry. An approximate (isotropic) treatment of cell e.s.d.'s is used for estimating e.s.d.'s involving l.s. planes.
Refinement. Refinement of *F*^2^ against ALL reflections. The weighted *R*-factor *wR* and goodness of fit *S* are based on *F*^2^, conventional *R*-factors *R* are based on *F*, with *F* set to zero for negative *F*^2^. The threshold expression of *F*^2^ > σ(*F*^2^) is used only for calculating *R*-factors(gt) *etc*. and is not relevant to the choice of reflections for refinement. *R*-factors based on *F*^2^ are statistically about twice as large as those based on *F*, and *R*- factors based on ALL data will be even larger.

### Fractional atomic coordinates and isotropic or equivalent isotropic displacement parameters (Å^2^)

**Table:**

	*x*	*y*	*z*	*U*_iso_*/*U*_eq_	
C1	0.7743 (2)	−0.1178 (3)	0.23967 (18)	0.0288 (4)	
C2	0.66925 (19)	−0.0224 (3)	0.15494 (15)	0.0272 (4)	
C3	0.6855 (2)	0.0055 (4)	0.03564 (15)	0.0304 (4)	
C4	0.5858 (3)	0.0965 (3)	−0.0557 (2)	0.0429 (6)	
H4	0.5020	0.1392	−0.0416	0.051*	
C5	0.6085 (3)	0.1243 (4)	−0.1655 (2)	0.0477 (6)	
H5	0.5400	0.1837	−0.2243	0.057*	
C6	0.7336 (3)	0.0636 (3)	−0.1883 (2)	0.0483 (6)	
H6	0.7504	0.0859	−0.2613	0.058*	
C7	0.8305 (3)	−0.0276 (4)	−0.10433 (19)	0.0445 (6)	
H7	0.9131	−0.0694	−0.1209	0.053*	
C8	0.8094 (2)	−0.0613 (3)	0.00897 (19)	0.0340 (5)	
C9	0.9098 (2)	−0.1605 (4)	0.0950 (2)	0.0418 (5)	
H9	0.9890	−0.2075	0.0756	0.050*	
C10	0.8947 (2)	−0.1897 (3)	0.2055 (2)	0.0382 (5)	
H10	0.9628	−0.2568	0.2595	0.046*	
C11	0.5465 (2)	0.0524 (3)	0.18248 (18)	0.0292 (4)	
H11	0.4778	0.1043	0.1198	0.035*	
C12	0.3897 (2)	0.1430 (3)	0.29272 (17)	0.0307 (4)	
H12	0.3676	0.2387	0.2349	0.037*	
C13	0.2635 (2)	0.0163 (5)	0.2663 (3)	0.0612 (9)	
H13A	0.2422	−0.0219	0.1851	0.092*	
H13B	0.1808	0.0738	0.2788	0.092*	
H13C	0.2879	−0.0829	0.3182	0.092*	
C14	0.4141 (2)	0.2180 (3)	0.41799 (18)	0.0274 (4)	
C15	0.7228 (3)	0.4372 (3)	0.3802 (3)	0.0555 (7)	
H15A	0.6852	0.3618	0.3132	0.083*	
H15B	0.7508	0.5467	0.3534	0.083*	
H15C	0.6501	0.4571	0.4202	0.083*	
C16	0.9720 (3)	0.2924 (4)	0.3760 (3)	0.0566 (7)	
H16A	1.0605	0.2349	0.4161	0.085*	
H16B	0.9930	0.3998	0.3418	0.085*	
H16C	0.9161	0.2180	0.3143	0.085*	
N1	0.52124 (17)	0.0554 (2)	0.28544 (14)	0.0268 (3)	
O1	0.77231 (16)	−0.1464 (2)	0.34856 (12)	0.0355 (3)	
O2	0.51906 (16)	0.1680 (2)	0.49919 (13)	0.0372 (4)	
O3	0.32074 (15)	0.3268 (2)	0.42825 (13)	0.0354 (3)	
O4	0.82733 (16)	0.1620 (2)	0.51567 (15)	0.0422 (4)	
S1	0.87374 (6)	0.33792 (8)	0.47907 (5)	0.03915 (13)	
Zn1	0.66361 (2)	−0.00892 (3)	0.440772 (16)	0.02749 (7)	

### Atomic displacement parameters (Å^2^)

**Table:**

	*U*^11^	*U*^22^	*U*^33^	*U*^12^	*U*^13^	*U*^23^
C1	0.0313 (10)	0.0256 (11)	0.0325 (9)	−0.0010 (8)	0.0141 (9)	−0.0017 (8)
C2	0.0308 (8)	0.0236 (10)	0.0288 (8)	0.0005 (10)	0.0113 (7)	−0.0007 (10)
C3	0.0376 (9)	0.0261 (11)	0.0303 (8)	−0.0042 (11)	0.0141 (8)	−0.0031 (10)
C4	0.0557 (15)	0.0403 (14)	0.0372 (11)	0.0075 (12)	0.0204 (12)	0.0064 (10)
C5	0.0727 (17)	0.0373 (14)	0.0331 (11)	−0.0006 (13)	0.0146 (12)	0.0055 (10)
C6	0.0787 (18)	0.0388 (13)	0.0374 (11)	−0.0155 (13)	0.0323 (14)	−0.0064 (10)
C7	0.0566 (13)	0.0462 (16)	0.0411 (10)	−0.0114 (14)	0.0308 (11)	−0.0094 (12)
C8	0.0387 (11)	0.0339 (13)	0.0330 (10)	−0.0073 (9)	0.0162 (9)	−0.0070 (8)
C9	0.0357 (11)	0.0521 (15)	0.0441 (12)	0.0053 (11)	0.0221 (11)	−0.0068 (11)
C10	0.0345 (11)	0.0424 (14)	0.0392 (11)	0.0104 (10)	0.0130 (10)	−0.0003 (10)
C11	0.0311 (10)	0.0269 (10)	0.0291 (9)	0.0025 (8)	0.0076 (9)	0.0001 (7)
C12	0.0280 (10)	0.0330 (12)	0.0319 (10)	0.0043 (9)	0.0096 (9)	−0.0071 (9)
C13	0.0308 (11)	0.073 (2)	0.0840 (18)	−0.0102 (14)	0.0225 (12)	−0.0487 (19)
C14	0.0279 (10)	0.0267 (10)	0.0333 (10)	−0.0044 (8)	0.0181 (9)	−0.0043 (8)
C15	0.0493 (14)	0.0333 (14)	0.088 (2)	0.0012 (11)	0.0255 (15)	0.0115 (12)
C16	0.0464 (15)	0.0558 (19)	0.0764 (19)	−0.0036 (13)	0.0319 (15)	−0.0019 (15)
N1	0.0266 (8)	0.0274 (9)	0.0284 (7)	0.0022 (6)	0.0106 (7)	−0.0035 (6)
O1	0.0414 (8)	0.0361 (9)	0.0325 (7)	0.0111 (7)	0.0161 (7)	0.0074 (6)
O2	0.0369 (8)	0.0455 (10)	0.0309 (7)	0.0056 (7)	0.0121 (7)	−0.0044 (7)
O3	0.0358 (8)	0.0372 (9)	0.0365 (7)	0.0056 (7)	0.0156 (7)	−0.0104 (7)
O4	0.0371 (8)	0.0357 (9)	0.0520 (9)	−0.0084 (7)	0.0094 (7)	0.0051 (8)
S1	0.0388 (3)	0.0311 (3)	0.0503 (3)	−0.0096 (2)	0.0169 (3)	−0.0059 (2)
Zn1	0.02884 (11)	0.02883 (11)	0.02707 (10)	0.00032 (12)	0.01157 (8)	0.00374 (11)

### Geometric parameters (Å, °)

**Table:**

C1—O1	1.300 (2)	C12—C14	1.531 (3)
C1—C2	1.410 (3)	C12—H12	0.9800
C1—C10	1.443 (3)	C13—H13A	0.9600
C2—C11	1.434 (3)	C13—H13B	0.9600
C2—C3	1.465 (2)	C13—H13C	0.9600
C3—C4	1.408 (3)	C14—O2	1.242 (3)
C3—C8	1.416 (3)	C14—O3	1.259 (3)
C4—C5	1.381 (3)	C15—S1	1.762 (3)
C4—H4	0.9300	C15—H15A	0.9600
C5—C6	1.391 (4)	C15—H15B	0.9600
C5—H5	0.9300	C15—H15C	0.9600
C6—C7	1.348 (4)	C16—S1	1.768 (3)
C6—H6	0.9300	C16—H16A	0.9600
C7—C8	1.422 (3)	C16—H16B	0.9600
C7—H7	0.9300	C16—H16C	0.9600
C8—C9	1.409 (3)	N1—Zn1	2.0119 (18)
C9—C10	1.361 (3)	O1—Zn1	2.0040 (15)
C9—H9	0.9300	O2—Zn1	2.1891 (16)
C10—H10	0.9300	O3—Zn1^i^	1.9560 (15)
C11—N1	1.296 (2)	O4—S1	1.5184 (18)
C11—H11	0.9300	O4—Zn1	2.0520 (17)
C12—N1	1.462 (2)	Zn1—O3^ii^	1.9560 (15)
C12—C13	1.521 (3)		
			
O1—C1—C2	124.91 (17)	C12—C13—H13B	109.5
O1—C1—C10	116.32 (19)	H13A—C13—H13B	109.5
C2—C1—C10	118.77 (17)	C12—C13—H13C	109.5
C1—C2—C11	121.86 (16)	H13A—C13—H13C	109.5
C1—C2—C3	119.89 (17)	H13B—C13—H13C	109.5
C11—C2—C3	118.24 (18)	O2—C14—O3	125.85 (18)
C4—C3—C8	116.96 (17)	O2—C14—C12	119.49 (17)
C4—C3—C2	124.27 (18)	O3—C14—C12	114.66 (19)
C8—C3—C2	118.77 (19)	S1—C15—H15A	109.5
C5—C4—C3	122.0 (2)	S1—C15—H15B	109.5
C5—C4—H4	119.0	H15A—C15—H15B	109.5
C3—C4—H4	119.0	S1—C15—H15C	109.5
C4—C5—C6	120.1 (2)	H15A—C15—H15C	109.5
C4—C5—H5	120.0	H15B—C15—H15C	109.5
C6—C5—H5	120.0	S1—C16—H16A	109.5
C7—C6—C5	119.9 (2)	S1—C16—H16B	109.5
C7—C6—H6	120.1	H16A—C16—H16B	109.5
C5—C6—H6	120.1	S1—C16—H16C	109.5
C6—C7—C8	121.5 (2)	H16A—C16—H16C	109.5
C6—C7—H7	119.2	H16B—C16—H16C	109.5
C8—C7—H7	119.2	C11—N1—C12	117.15 (17)
C9—C8—C3	119.56 (17)	C11—N1—Zn1	125.29 (13)
C9—C8—C7	120.97 (19)	C12—N1—Zn1	116.50 (12)
C3—C8—C7	119.5 (2)	C1—O1—Zn1	126.51 (14)
C10—C9—C8	122.14 (19)	C14—O2—Zn1	114.09 (12)
C10—C9—H9	118.9	C14—O3—Zn1^i^	126.85 (14)
C8—C9—H9	118.9	S1—O4—Zn1	134.27 (11)
C9—C10—C1	120.8 (2)	O4—S1—C15	108.15 (11)
C9—C10—H10	119.6	O4—S1—C16	106.05 (12)
C1—C10—H10	119.6	C15—S1—C16	98.14 (15)
N1—C11—C2	126.87 (19)	O3^ii^—Zn1—O1	98.54 (7)
N1—C11—H11	116.6	O3^ii^—Zn1—N1	138.57 (7)
C2—C11—H11	116.6	O1—Zn1—N1	88.32 (7)
N1—C12—C13	111.01 (19)	O3^ii^—Zn1—O4	101.12 (7)
N1—C12—C14	108.94 (16)	O1—Zn1—O4	96.09 (7)
C13—C12—C14	109.48 (17)	N1—Zn1—O4	118.82 (7)
N1—C12—H12	109.1	O3^ii^—Zn1—O2	93.89 (7)
C13—C12—H12	109.1	O1—Zn1—O2	166.10 (6)
C14—C12—H12	109.1	N1—Zn1—O2	78.22 (6)
C12—C13—H13A	109.5	O4—Zn1—O2	87.52 (7)
			
O1—C1—C2—C11	−0.5 (4)	C13—C12—N1—C11	89.0 (2)
C10—C1—C2—C11	179.8 (2)	C14—C12—N1—C11	−150.37 (18)
O1—C1—C2—C3	178.2 (2)	C13—C12—N1—Zn1	−102.1 (2)
C10—C1—C2—C3	−1.4 (3)	C14—C12—N1—Zn1	18.5 (2)
C1—C2—C3—C4	179.0 (2)	C2—C1—O1—Zn1	−20.7 (3)
C11—C2—C3—C4	−2.2 (4)	C10—C1—O1—Zn1	158.98 (16)
C1—C2—C3—C8	−1.5 (3)	O3—C14—O2—Zn1	−175.97 (16)
C11—C2—C3—C8	177.3 (2)	C12—C14—O2—Zn1	4.9 (2)
C8—C3—C4—C5	−1.7 (4)	O2—C14—O3—Zn1^i^	16.7 (3)
C2—C3—C4—C5	177.8 (3)	C12—C14—O3—Zn1^i^	−164.18 (13)
C3—C4—C5—C6	−0.8 (4)	Zn1—O4—S1—C15	23.79 (18)
C4—C5—C6—C7	2.2 (4)	Zn1—O4—S1—C16	−80.64 (17)
C5—C6—C7—C8	−1.1 (4)	C1—O1—Zn1—O3^ii^	165.79 (17)
C4—C3—C8—C9	−177.1 (2)	C1—O1—Zn1—N1	26.85 (17)
C2—C3—C8—C9	3.4 (3)	C1—O1—Zn1—O4	−91.96 (18)
C4—C3—C8—C7	2.8 (3)	C1—O1—Zn1—O2	12.5 (4)
C2—C3—C8—C7	−176.7 (2)	C11—N1—Zn1—O3^ii^	−122.75 (17)
C6—C7—C8—C9	178.4 (2)	C12—N1—Zn1—O3^ii^	69.40 (17)
C6—C7—C8—C3	−1.4 (4)	C11—N1—Zn1—O1	−21.72 (17)
C3—C8—C9—C10	−2.4 (4)	C12—N1—Zn1—O1	170.43 (14)
C7—C8—C9—C10	177.7 (2)	C11—N1—Zn1—O4	74.31 (18)
C8—C9—C10—C1	−0.6 (4)	C12—N1—Zn1—O4	−93.54 (15)
O1—C1—C10—C9	−177.2 (2)	C11—N1—Zn1—O2	154.80 (18)
C2—C1—C10—C9	2.5 (3)	C12—N1—Zn1—O2	−13.05 (14)
C1—C2—C11—N1	5.6 (4)	S1—O4—Zn1—O3^ii^	−170.20 (14)
C3—C2—C11—N1	−173.2 (2)	S1—O4—Zn1—O1	89.82 (14)
N1—C12—C14—O2	−15.1 (3)	S1—O4—Zn1—N1	−1.61 (17)
C13—C12—C14—O2	106.5 (2)	S1—O4—Zn1—O2	−76.72 (14)
N1—C12—C14—O3	165.76 (17)	C14—O2—Zn1—O3^ii^	−134.50 (15)
C13—C12—C14—O3	−72.7 (3)	C14—O2—Zn1—O1	19.0 (4)
C2—C11—N1—C12	178.6 (2)	C14—O2—Zn1—N1	4.39 (14)
C2—C11—N1—Zn1	10.9 (3)	C14—O2—Zn1—O4	124.51 (15)

Symmetry codes: (i) −*x*+1, *y*+1/2, −*z*+1; (ii) −*x*+1, *y*−1/2, −*z*+1.
